# Activity-Based Interventions to Increase Independence After Stroke In the Hospital Setting: Protocol for a Systematic Review

**DOI:** 10.2196/73133

**Published:** 2025-07-08

**Authors:** Tayla Grant, Laura Jolliffe, Kylie Wales, Emma Schneider, Avril E Drummond, Natasha A Lannin

**Affiliations:** 1 Monash University Melbourne Australia; 2 Alfred Health Melbourne Australia; 3 Peninsula Health Frankston Australia; 4 National Centre for Healthy Ageing Melbourne Australia; 5 Australian Catholic University North Sydney Australia; 6 Department of Allied Health Swinburne University of Technology Melbourne Australia; 7 University of Nottingham Nottingham United Kingdom

**Keywords:** stroke, rehabilitation, activities of daily living, occupational therapy, stroke rehabilitation, hemiplegia

## Abstract

**Background:**

Stroke is a leading cause of disability, commonly resulting in difficulty completing basic and instrumental activities of daily living. Rehabilitation is recommended to improve functional performance; however, it remains unknown whether early (hospital-based) intervention is effective; nor is it known what intervention components should be delivered.

**Objective:**

We propose a systematic review to summarize the evidence for the effectiveness of activity-based interventions, led by occupational therapists, for improving performance of basic activities of daily living (b-ADL) or simple cognitive instrumental activities of daily living (C-IADL) among adults with stroke. Reviewing simple C-IADLs as opposed to instrumental activities of daily living will allow us to review interventions for activities with similar executive functioning demands. By identifying effective interventions, we aim to improve outcomes for stroke survivors by maximizing independence during hospitalization.

**Methods:**

Searches will be conducted in MEDLINE, CINAHL, EMBASE, and Cochrane Central Register of Controlled Trials. We will include randomized controlled trials and quasi-randomized controlled trials that include adult stroke survivors and tested interventions (delivered in the hospital setting) aiming to improve or promote independence in b-ADLs or C-IADLs. Two reviewers will independently screen full-text articles, and one reviewer will extract data, with a second reviewer providing confirmation. The Physiotherapy Evidence Database (PEDro) scale will be used to assess the methodological quality of studies. The Cochrane *Q* test will assess heterogeneity among studies, and where appropriate, meta-analysis will then be performed. Measures of outcomes may include global ratings of function (such as the Functional Independence Measure) or specific task performance assessments (such as the Nottingham Dressing Assessment).

**Results:**

Results will be presented based on subgroup analyses where possible, including activity type (activity or occupation level), time after stroke (within or after 1 week), and delivery of intervention (group or individualized).

**Conclusions:**

This systematic review will address the current gap in the literature regarding activity-based interventions for stroke survivors in the inpatient setting and provide clinical guidance on the most effective methods, if any, for improving independence early after stroke.

**Trial Registration:**

PROSPERO CRD42024562195; https://www.crd.york.ac.uk/PROSPERO/view/CRD42024562195

**International Registered Report Identifier (IRRID):**

DERR1-10.2196/73133

## Introduction

Stroke is a leading cause of adult disability [1]; despite advances in acute treatments, the need for neurorehabilitation continues [2,3]. Stroke-related impairments can lead to difficulties in performing meaningful activities, such as showering or preparing a meal, and problems in participating more broadly in life, such as maintaining a home. Previous studies have shown that around two-thirds of all survivors of stroke experience a disability that impacts their ability to complete activities of daily living (ADLs) [4]. ADLs are the everyday tasks completed by an individual and are often divided in multiple domains, including basic activities of daily living (b-ADLs) and simple and complex cognitive instrumental activities of daily living (C-IADLs). Basic ADLs “rely heavily on physical abilities and procedural memory” [5], such as self-feeding, dressing, grooming, and showering, whereas simple C-IADLs are tasks that “are more familiar, contain fewer steps and require less planning or problem solving” [5], such as preparing a hot drink, preparing a simple meal, or folding clothing. Complex C-IADLs are “more novel, less predictable, and place greater demands on multitasking, planning, decision making, and problem-solving capacities” [5], such as organizing and managing medications, planning an event, or creating a budget for an extended period of time.

Effective rehabilitation enables stroke survivors to achieve optimal functional outcomes across all areas of their life [4]. Occupational therapists assess and deliver rehabilitation for stroke survivors who have experienced changes to their ability to engage in meaningful everyday activities as a result of their stroke impairments [6]. Occupational therapy interventions often target ADLs to improve functional performance [7].

Stroke survivors should receive intensive rehabilitation throughout their inpatient stay to support a meaningful recovery [4] and promote neuroplasticity [3]. Neuroplasticity is the theory that neural pathways are able to adapt and regenerate to improve function [8]. Neuroplasticity is time sensitive, and research has shown that early rehabilitation is linked to improved functional recovery [9,10]. Previous research has explored the effectiveness of occupational therapy rehabilitation among stroke survivors for ADLs in a community setting [11]. The results highlighted that stroke survivors who received ADL-targeting occupational therapy in the community had increased levels of ADL independence [11]. There were limitations inherent in this systematic review; in particular, only a third of previous trials used appropriate randomization and allocation concealment methods [11]. Overall, research on the effects of activity-based interventions alone in the inpatient setting has been limited [12]. Examining the available evidence, a recent systematic review found that occupational therapy–specific interventions in the inpatient setting did not have a significant effect on improving quality of life and activity performance of stroke survivors [13]. These results should be interpreted cautiously given the substantial heterogeneity of the IADL performance analysis (*I*^2^ of 70.69) and that both the control and experimental groups were provided occupation-specific interventions, which does not allow the reader to discern the effectiveness of activity-based interventions from this systematic review [13]. Additional evidence from an observational study suggests that intensive hospital rehabilitation correlates with improved ADL performance on discharge. However, the specific type of rehabilitation completed in this study was unclear [12]. The literature on the effectiveness of occupational therapy specifically for stroke survivors in the hospital setting is limited, and studies to date have recommended further research to determine the specific rehabilitation interventions and intensity required [12,14].

Therefore, this systematic review will synthesize activity-based intervention studies that aim to improve performance of b-ADLs and/or simple C-IADLs in the hospital setting among stroke survivors. Complex C-IADLs were excluded from this review as the demands of these tasks were identified as being significantly different from b-ADLs and simple C-IADLs. The guiding research question developed was as follows: Do occupational therapy–led, activity-based interventions designed to target b-ADLs and/or simple C-IADLs in the hospital setting increase functional independence in stroke survivors?

## Methods

### Study Design

The protocol was developed according to the Preferred Reporting Items for Systematic Reviews and Meta-Analyses (PRISMA) Protocols statement [15]; the PRISMA statement will guide reporting of the completed systematic review. The protocol has been registered in the International Prospective Register of Systematic Reviews (PROSPERO; CRD42024562195).

### Eligibility Criteria

The primary objective of this review is to synthesize effectiveness research on rehabilitation interventions delivered in the hospital setting to improve independence after stroke. We will include publications from any date in our search.

#### Types of Studies

Studies will be included if they are full publications of randomized controlled trials (RCTs) and quasi-RCTs.

#### Population and Setting

Studies that include adults (aged ≥18 years) diagnosed with stroke who are inpatients in the hospital setting will be included. Studies involving mixed diagnoses will be included where the data from those with a diagnosis of stroke are able to be extracted, or where ≥75% of the participants have a diagnosis of stroke. Studies involving mixed age groups will be included where the data from those aged ≥18 years are able to be extracted, or where ≥75% of the participants are aged ≥18 years. Studies will be excluded if they were performed with people who reside in the community setting, including care-based facilities (eg, residential aged care).

#### Intervention

Studies that provide an activity-based intervention completed in the hospital setting and that fit within the scope of occupational therapy will be reviewed. Retraining interventions specific to b-ADLs and simple C-IADLs will be included in our systematic review. Both compensatory and remedial rehabilitation strategies will be included. We will exclude community-based interventions and computer-based or virtual reality interventions given the focus of the systematic review on the effects of real-life training in the hospital environment. For studies that include a combination of activity-based interventions alongside other interventions, studies will be included where the effects can be isolated (ie, the only difference between the experimental group and the comparator group is the activity-based intervention).

#### Comparison Interventions

Included studies may have either an inactive comparison (where the control group received no occupational therapy input) or an active comparison, such as those where participants received an occupational therapy intervention that was not specific to activity-based retraining for ADLs or where a nondescriptive intervention was used in the control group. Examples of this include impairment-based treatments, such as upper limb treatment or visual scanning retraining.

#### Outcomes

We will record the outcome measures that the included studies used to report outcomes related to b-ADLs and simple C-IADLs to determine the effectiveness of activity-based interventions on stroke survivors’ functional independence. All clinical measures used to evaluate the effectiveness of activity-based interventions will be considered. Outcome measures may be reported as a global measure of function, such as the Functional Independence Measure, or specific evaluations of b-ADLs or simple C-IADLs, such as the Nottingham Dressing Assessment or Barthel index.

Textbox 1 provides a summary of the eligibility criteria that will shape the search terms and identify relevant studies.

**Eligibility criteria.**

**Inclusion criteria**

- Participants: at least 75% aged 18 years or older with a diagnosis of stroke
- Intervention: activity-based retraining (basic activities of daily living [b-ADLs] or simple cognitive instrumental activities of daily living [c-IADLs]); intervention delivered in inpatient setting
- Comparison: no occupational therapy input, no allied health, occupational therapy not specific to activity-based retraining for activities of daily living, or intervention not defined in the control group
- Outcomes: (1) determine the effectiveness of activity-specific interventions (b-ADLs and/or simple C-IADLs) on stroke survivors’ functional independence; (2) identify and describe the assessments and occupational therapy interventions designed to target b-ADLs and simple C-IADLs currently being used with adult stroke survivors in the hospital setting
- Study design: randomized controlled trials and quasi-randomized controlled trials

**Exclusion criteria**

- Intervention: computer-based or virtual reality retraining; community-based interventions

### Search Strategy

Electronic searches will be conducted in CINAHL, EMBASE (via Ovid) and MEDLINE. The search strategy will be developed with senior researchers. The search strategy will include key terms such as *stroke*, *hemiplegia*, *activities of daily living*, *activity-based interventions*, and *hospital*. During screening of titles and abstracts, only English-language articles will be included due to no access to translation.

### Procedures

All studies identified by the search strategy will be uploaded to Covidence (Veritas Health Innovation Ltd), and duplicates will be removed. All articles will be screened at the title and abstract stage by one reviewer, in line with the exclusion and inclusion criteria. Duplicate studies (ie, those with the same authors, setting, location, and interventions) will be removed. All articles excluded at the title and abstract screening stage will be reviewed by a senior researcher to ensure no articles were inadvertently excluded. Full-text articles will then be reviewed by 2 research team members. If there are conflicts in decision-making, a third research team member will be consulted for a third review. Authors of studies may be contacted if there is missing information or further details are required to ensure articles are not inadvertently excluded or included. Details of the data screening will be presented in a PRISMA study flow chart.

One reviewer (TG) will extract data using a predetermined form; all data extraction will be confirmed by the senior author (NAL). If discrepancies occur, these will be resolved by discussion between the authors. If the discrepancies are not resolved, a third author will independently extract the data. The data extraction form will extract variables necessary for describing the study and conducting data analysis, including the following: the title of the paper, the authors’ names, the year the study was completed, the study design (eg, RCT or quasi-RCT), the country where the study was completed, participant age and sex, time after stroke of participants, primary and secondary assessments used, intervention and comparison details (according to the Template for Intervention Description and Replication [TIDieR] checklist items [16]), and the outcomes and results (eg, number of participants in analysis; summary data for each intervention group; estimate of effect, along with measure of variability, eg, mean difference; and 95% CI around the estimate).

The Physiotherapy Evidence Database (PEDro) scale will be used to critically appraise the studies to determine any risk of bias [17]. The PEDro scale scores the methodological quality of clinical trials [18]. It is an 11-point scale exploring the internal and external validity of studies and is known to be reliable for use in systematic reviews [17]. The OTseeker database will be used, if available; however, if not available online, PEDro will be completed by 2 reviewers separately. If there are disagreements regarding the results, the 2 reviewers will first attempt to resolve them between themselves; if they are unable to reach a consensus, a third reviewer who is a senior researcher in the research team will review and make the final decision.

### Statistical Analysis

A meta-analysis will be completed to compare effects between treatment and control groups. RevMan will be used to conduct a statistical analysis to calculate effect measures. The treatment effect measure will be determined by the data located. For instance, continuous data may be calculated using the standard mean difference and 95% CI when studies measure the same concept but have used a different measurement scale. The mean difference and 95% CI may be used when the results are expressed using the same measurement scale. The impact of heterogeneity will be reviewed. If results are too dissimilar, a sensitivity analysis will be considered and a narrative synthesis will be considered as an alternative.

## Results

Results will be discussed based on subgroup analyses where possible, including activity type (activity or occupation level), time after stroke (within or after one week), and delivery of intervention (group or individualized). Data extraction and analysis were completed in February 2025, and the results of the study and the submission of a manuscript for peer review are expected in July 2025.

## Discussion

### Overview

The findings from this systematic review will aim to address the gap in the literature regarding activity-based interventions for stroke survivors in the inpatient setting. Clinicians will be able to use the results to make evidence-informed decisions regarding activity-based interventions and rehabilitation for reducing long-term disability.

### Limitations

This review will only search for and synthesize studies published in English; this limitation will be acknowledged in the systematic review when it is published. We acknowledge that the heterogeneity of the intervention types and of the clinical measures used to evaluate independence may not permit direct comparisons between studies. These limitations are not uncommon in systematic reviews of complex interventions.

### Comparison With Prior Work

Prior reviews [13] did not systematically synthesize intervention effects from only those studies that delivered activity-based interventions with the goal of increasing independence. Findings will therefore allow reliable conclusions regarding such interventions to be determined. Consistent with the prior work, we will use a validated tool (PEDro) to assess the quality of evidence.

### Conclusion

This systematic review will provide a detailed summary of the current evidence for the effectiveness of activity-based interventions on functional independence for stroke survivors. The findings of this review will guide clinicians in their selection of rehabilitation interventions and enable researchers to determine future evidence needs. Synthesis of published effectiveness trials will not only support evidence-based practice from an occupational therapy perspective, but will also guide future trial design, given the limited overall research available to date in the inpatient setting.

## Abbreviations

ADL: activity of daily living
b-ADL: basic activity of daily living
C-IADL: cognitive instrumental activity of daily living
PEDro: Physiotherapy Evidence Database
PRISMA: Preferred Reporting Items for Systematic Reviews and Meta-Analyses
PROSPERO: International Prospective Register of Systematic Reviews
RCT: randomized controlled trial
TIDieR: Template for Intervention Description and Replication

## Data Availability

The data generated and analyzed during this study will include content extracted from published, peer-reviewed journal articles. Additional data generated and analyzed during the study will be available from the corresponding author upon reasonable request.
